# A Case of Suppuration in the Omentum, Succeeded by a Pneumonic Inflammation

**Published:** 1803-07-01

**Authors:** W. Robertson

**Affiliations:** one of the Surgeons to the Kelso Dispensary


### *A* Case *of* Suppuration *in the* Omentum, *succeeded by a* Pneumonic Inflammation; *by* W. Robertson, *one of the Surgeons to the Kelso Dispensary.*

MR. THOMAS D. aged 47, of an athletic make and full habit of body, by business a farmer; on the 18th of August, 1802, while in the field attending his labourers, was seized with a violent pain above the right ilea, extending up that side to the epigastic region, which continued so severe upwards of an hour, that he had great difficulty in getting home. After he had been some time in the house, the violence of the pain abated.

On the 6th of September he called on me, and stated the above particulars. The origin of his complaint he imputed to cold, caught in consequence of his having changed his flannel drawers, which he had been in the habit of wearing, for cotton ones on that day. He mentioned to me, that though following his ordinary employment, he found himself unwell; and, from that date, he complained of a stiffness attended with some degree of pain in the abdomen, concerning which he wished to take my advice.

At that time I could perceive nothing materially wrong, either in the state of the pulse, or in the part complained of. As he was of a plethoric habit of body, I suggested to him the propriety of his losing a little blood and taking some cooling cathartic medicine, to which he readily agreed. I took about twelve ounces of blood from the arm, upon which

---

which there was no size, and administered two doses of physic, composed of the pulv. jalap. compos. ℈ij. calomel, pt. gr. v. M. each, to be taken at proper intervals; by which, if his complaint was not relieved, to let me know.

I heard nothing of him till the 17th of September, when he called, and told me that he found the stiffness in the abdomen increasing, accompanied with a swelling that gave him great pain whenever he moved himself. Upon a careful examination of the part complained of, I discovered a hard tumour, in size nearly equal to a goose egg, deeply seated a little above the umbilicus in the lower part of the epigastic region, inclining a little to the right side, which gave pain when pressed. I mentioned to him, that a gentle mercurial course, with a strict adherence to a cooling regimen, was the only means hitherto found useful in discussing these indurated tumours. But as I could not pretend to say what might be the termination of such a swelling, for his own satisfaction, I wished him to take the advice of some other medical person, to which he assented. That gentleman, in addition to what I had prescribed, was of opinion that hot baths of sea water might have a tendency to expedite the discussion. He was also ordered to rub about a scruple of the ung. merc. fort. into the part affected twice a day, to use the baths every day, or every other day, as he found it agree with him, and to take a cup full of salt water every morning to keep his bowels regular. During the use of these prescriptions, he passed an uncommon quantity of urine of a very turbid nature, highly loaded with a lateritious substance. The mercury, in seven days, affected his mouth so much as to bring on such a degree of salivation as obliged him to discontinue it. He went ten times into the bath; but finding himself much weaker, and at times subject to fainting, his complaint still increasing, he returned home, after being absent a fortnight.

I visited him the day following, and found the tumour not only increasing but also occasioning a great deal of pain. He was likewise seized with a nausea, which did not allow any kind of food to remain on the stomach. He was ordered to take the saline effervescent mixture, and a number of leeches were applied over the surface of the tumour, alternated with poultices composed of hemlock, oatmeal, vinegar, and camphorated oil. As no aperient medicine would sit upon his stomach, his bowels were kept open by emollient injections. He was also ordered to live upon a light nourishing diet. Notwithstanding the use of these discussive measures, the tumour still continued to increase crease in size and hardness. His case now appeared very alarming. Mr. Russell, Surgeon, of Edinburgh, was desired to visit him. At that time the tumour occupied most of the epigastic region, inclining considerably to the right hypochondriac. The tumour, both in situation and point of feeling, had assumed the appearance of a diseased schirrous liver, although it had none of the other concomitant symptoms of that complaint. After a very minute examination, Mr. Russell could not say whether it was of a schirrous nature, or might terminate in a suppuration. The patient was directed to take daily two drachms of Rochel salts dissolved in a cup full of water, and an anodyne at bed time, both of which his stomach rejected. As the tumour was still increasing in size, the hemlock poultices were exchanged for emollient ones, which were frequently renewed and alternated with warm fomentations. At last, an obscure fluctuation was observed, and it became a little prominent about half in inch above the umbilicus, inclining rather to the left side, where an opening was made of more than an inch in length on the 26th of October, by the advice and in presence of Mr. Benjamin Bell, Surgeon in Edinburgh, and Dr. Douglas, Physician, Kelso. From the opening, about a pint and a half of a very fœtid matter was discharged. The state of the parts was examined by introducing the finger into the orifice, and, as far as it could reach, the omentum appeared diseased and indurated, with sinuses running in different directions. The wound was dressed and an emollient poultice applied over the whole. During the course of the disease, the pulse never seemed to deviate from its natural state, in proportion to the violence of the disorder. The patient being very much emaciated, was desired to live on a full diet, consisting chiefly of rich soups, and to take a certain quantity of wine daily. Half a drachm of the pulv. cort. peruv. among two ounces of the saline effervescent mixture, with four drops of L. L. to cause it to sit upon his stomach, every two hours was ordered. A considerable quantity of matter was discharged every time the wound was dressed, but a very great degree of hardness still remained.

In the course of a fortnight his recovery was very rapid. From being unable to move himself, in that time he acquired so much strength as to walk across the room unassisted. The discharge soon began to diminish, although every mean was used to keep the wound open, both by tents and the application of warm poultices. The part affected fected then began to enlarge, accompanied with pains similar to the former, and assumed the appearance of a fresh suppuration.

On the 22d of November, he was attacked with a violent pain in the left side, about the middle of the false ribs, which struck across his breast, and sometimes to his back, attended with cough, hæmoptoe, and great difficulty of breathing. His pulse being full, he was immediately blooded. Sixteen ounces were taken from his arm, which was exceedingly sizy. He had a cooling cathartic composed of Rochel salts and manna. A large blister was applied to the affected part. The pain shifted higher in his breast. The blooding was frequently repeated. In six days he lost four pounds four ounces, all sizy. He had four large blisters applied to the different parts of his chest before the pain and difficulty of breathing were relieved. He was ordered to take daily a drachm of nitre with two of cream of tartar dissolved among a pint and a half of thin gruel; his bowels to be kept open by taking a cup full of an infusion of senna, manna, and tamarinds, every night at bed time. He was allowed for diet nothing but panada, gruels, tea, and vegetables. By the evacuations, he was so much reduced that he could not be moved for eight days, without being threatened with fits of syncope.

After the pains abated, he still continued the use of the nitre and cream of tartar for the space of six weeks, and along with them a pill, consisting of half a grain of calomel every night at bed time. Under this mode of treatment he began to recover gradually, though slowly. He was much distressed with a teazing cough, for which he was ordered the use of ass-milk. By continuing it for a length of time, the cough left him. During this last illness, the former complaint of the abdomen was but little attended to, as his situation in other respects appeared so alarming, that his life was, in a great measure, despaired of. The great degree of hardness that was felt before the attack of the pulmonic affection, began gradually to diminish. The wound was kept open as long as possible; it, however, at last closed; at present there is scarcely any of the hardness remaining.

He now follows his usual employment, appears healthy, and has acquired his former fulness of habit. He has not, however, ventured upon any other diet than broths, milk, and vegetables. Nothing of his complaints now remains, except, at times, a difficulty of breathing. Whether this is occasioned by dyspepsia, or by an enlargement of some of the abdominal viscera, time alone can determine.

